# Ill Effects and Complications Associated to Removable Dentures With Improper Use and Poor Oral Hygiene: A Systematic Review

**DOI:** 10.7759/cureus.28144

**Published:** 2022-08-18

**Authors:** Amulya Dakka, Zahra Nazir, Humaira Shamim, Marie Jean, Muaaz Umair, Pratyusha Muddaloor, Michelle Farinango, Akhil Ansary, Safeera Khan

**Affiliations:** 1 Internal Medicine, California Institute of Behavioral Neurosciences & Psychology, Fairfield, USA; 2 Internal Medicine Clinical Research, California Institute of Behavioral Neurosciences & Psychology, Fairfield, USA; 3 Dermatology, California Institute of Behavioral Neurosciences & Psychology, Fairfield, USA; 4 Psychiatry, California Institute of Behavioral Neurosciences & Psychology, Fairfield, USA; 5 Research, California Institute of Behavioral Neurosciences & Psychology, Fairfield, USA; 6 General Medicine, California Institute of Behavioral Neurosciences & Psychology, Fairfield, USA; 7 Clinical Sciences, St. Martinus University Faculty of Medicine, Willemstad, CUW

**Keywords:** aspiration pneumonia, oral diseases, oral health care, removable denture, poor oral hygiene

## Abstract

The importance of oral care and its relationship with an individual’s well-being has been identified over the past few decades. As there is a drastic increase in the aging population, so did the use of removable dentures more than ever before. The use of dentures among the elderly provides functional advantages and esthetic benefits. However, improper use and poor oral hygiene with removable dentures come with complications, including denture stomatitis, ulcerations, pneumonia, and many more. The study aims to determine the complications associated with inappropriate use and poor oral care and bring forth evidence-based dentist-recommended guidelines for denture maintenance.

Articles were systematically screened in PubMed/Medline (Medical Literature Analysis and Retrieval System Online), PubMed Central (PMC), and Cochrane Library using keywords. Medical Subject Heading (MeSH) was also utilized to identify relevant articles. Inclusion and exclusion criteria were applied, duplicate articles were discarded, and then the articles were reviewed by title and abstract screening. The remaining articles went through a detailed full-text review. A quality appraisal check was conducted for each unique type of research publication, after which a total of 22 articles were finalized.

In this study, we have seen pathological biofilm formation on dentures, life-threatening pneumonia, denture stomatitis, and accidental ingestion/aspiration of dentures amongst the elderly population. The study also identified members with low literacy rates, minorities, and low-income families seem to be at higher risk of poor oral care and denture hygiene. We identified that most of these complications could be prevented with proper guidance and education. In the future, a further detailed study is important as no clear consensus exists in terms of best practices of denture cleaning methods. In addition, measures should be initiated to encourage regular dentist appointments and increase accessibility among members of low socioeconomic status and minorities.

## Introduction and background

In 2019, approximately 16.5% of the American population was 65 years or older; by 2050, this percentage will rise to 22% [1]. One aspect of aging that affects almost all the elderly population is edentulism (lack of teeth). The ratio of edentulous to non-edentulous people is roughly 2:1. About 23 million people completely miss teeth, while another 12 million are missing teeth in one arch. Ninety percent of people with edentulism have dentures, whether fixed or removable [2]. An arch of prosthetic teeth anchored firmly to artificial gum tissue on top of the natural gum line is known as a fixed denture [3]. Complete and partial removable dentures are the two types of removable dentures. Cast metal framework, acrylic clasp, and flexible partial dentures make up removal dentures inserted into a gum-colored mold on top of the natural gums. Complete dentures are full-arch oral prosthetics that replace all teeth in a missing arch [3-4].

In the elderly population, dentures provide functional and cosmetic benefits. However, wearing dentures comes with some risks: ill-fitting dentures, incorrect use, and poor oral hygiene can result in irritation of the gums and oral mucosa, ulcerations, and denture stomatitis [5]. Denture surfaces have also been identified as a reservoir for various pathogenic organisms. Aspiration pneumonia, halitosis, infective endocarditis, septic meningitis, and other complications can result from biofilm formation. In addition, accidental aspiration and ingestion of dentures are also more common in the elderly; potential risk factors include alcoholic intoxication, advancing dementia, stroke, and epilepsy [6]. Necrosis, perforation, ulceration, fistula formation, penetration of neighboring organs, bleeding, and obstruction are risks associated with ingesting dentures [6-7]. 

These clinical situations and life-threatening complications may be merely prevented with proper guidance from medical professionals, including primary care providers, dentists, and dental hygienists. Even though more education is required, relatively little information is accessible about appropriate, evidence-based techniques for cleaning dentures and their optimal use [8]. According to studies, at least 40% of senior patients do not disinfect properly or remove their dentures at night, and serious pneumonia occurrences are twice as frequent in these individuals [9]. Hence, professional advice on proper denture use and cleaning is essential for all users, particularly the elderly and their caregivers [8]. 

The lack of proper education among denture users and the lack of routine dental care services among the underserved and elderly worsen the problem further [9]. Many older individuals cannot pay for the preventive and rehabilitative care they require [9]. Improving patients' oral health literacy personally may also be necessary to avoid and prevent future issues [8]. 

We conducted a systematic study to assess the complications, infections, and life-threatening illnesses due to inappropriate denture use and poor dental hygiene in the geriatric population who wear removable dentures. We also analyzed factors contributing to worsening oral health among the elderly such as diabetics, alcohol, and tobacco use [10-11]. By the end of the review, we aim to bring further proper dentist-recommended evidence-based denture oral hygiene and appropriate use of dentures.

## Review

Method 

This systemic review was designed, and its data were reported using the principles of the Preferred Reporting Items for Systemic Review and Meta-Analysis (PRISMA) 2020 Guideline [12]. 

Search Strategy 

A computer-assisted search was performed in the medical databases PubMed, PubMed Central (PMC), MEDLINE, and Cochrane Library from 12/14/2021 to 1/14/2022. Multiple keyword combinations were used in the search, including “Dentures and complications,” “Dentures and halitosis,” “Dentures and Pneumonia,” “Dentures and stomatitis,” “Dentures and ulcerations,” “Dentures and infections,” and “Dentures and oral hygiene.” MeSH (Medical Subject Heading) search strategy was also utilized: ("Denture, Partial, Removable/adverse effects"[Mesh] OR "Denture, Partial, Removable/classification"[Mesh] OR 

"Denture, Partial, Removable/etiology"[Mesh] OR "Denture, Partial, Removable/microbiology"[Mesh] OR 

"Denture, Partial, Removable/pharmacology"[Mesh] OR "Denture, Partial, Removable/psychology"[Mesh] OR 

"Denture, Partial, Removable/virology"[Mesh]). We used “Dentures and infections or complications or ulcerations or stomatitis or pneumonia or halitosis or oral hygiene” to search for articles in Cochrane Library. Tables 1-2 show the search results.

**Table 1:** Search results from PubMed, PMC, and MEDLINE. PMC, PubMed Central

PubMed, PMC, and MEDLINE	Total search results	After inclusion and exclusion criteria
"Dentures and infections"	2087	57
"Dentures and complications"	3912	82
"Dentures and oral hygiene"	2192	79
"Dentures and stomatitis"	1697	27
"Dentures and ulcerations"	268	13
"Dentures and pneumonia"	114	16
"Dentures and halitosis"	66	3
"Denture, Partial, Removable/adverse effects"[Mesh] OR “Denture, Partial, Removable/classification"[Mesh] OR “Denture, Partial, Removable/etiology"[Mesh] OR "Denture, Partial, Removable/microbiology"[Mesh] OR "Denture, Partial, Removable/pharmacology"[Mesh] OR "Denture, Partial, Removable/psychology"[Mesh] OR "Denture, Partial, Removable/virology"[Mesh]”	608	14
Total	10944	294

**Table 2:** Search results from Cochrane Library.

Cochrane Library	Total search results
"Dentures and infections or complications or halitosis or pneumonia or ulcerations or stomatitis or oral hygiene"	1668

Inclusion and Exclusion Criteria 

We selected only articles published from 2012 to 2022, written in English, studies with patients 65 years or older, and global publications were chosen, and only articles with human studies were included. Articles were excluded if they were required to be purchased or if they were gray literature, they did not directly correlate to the research objectives, and if only abstracts were available. 

Selection Process 

All articles obtained by search strategy were transferred to the Endnote application, and duplicate articles were excluded with the assistance of the same. Each article was screened by title and abstracts, independently assessed by the first and second authors' AD and ZN. Any conflicts regarding the eligibility of articles were discussed, and inclusion and exclusion criteria were applied before reaching a mutual agreement. All the remaining articles were further evaluated by reviewing the full text to exclude irrelevance. 

Data Collection Process 

Once the final articles were selected for the systemic review, all the authors listed were equally involved in assessing each article for quality check and retrieving necessary information to be included in the review article. 

Quality Assessment of the Studies

The randomized clinical trials were critically appraised with the Cochrane Risk of Bias Assessment Tool 2 [13], while the systematic reviews were evaluated by the Assessment of Multiple Systematic Review (AMSTAR) tool [14]. Along with these, the Joanna Briggs Institute (JBI) critical appraisal checklist was applied for case reports [15], and the Newcastle Ottawa Quality Assessment tool was used for observational studies [16]. Lastly, the scale for the Assessment of Narrative Review (SANRA) was utilized for publications with no specific method mentioned [17]. Studies with a quality appraisal score greater than 60% were included in the systematic review.

Results 

Study Identification and Selection

After an extensive search, 12612 articles were obtained with the above search criteria. There were 10944 publications from PubMed, PMC, and Medline, and 1668 articles from Cochrane Library. Endnote website application was used to remove duplicate articles. Out of those, 10188 articles remain after removing 2424 duplicate articles. Then inclusion and exclusion criteria were applied, through which 8518 were excluded. At this point, 1670 articles remain to be evaluated further through title, abstract and full-text analysis. Hence, 37 articles remained after the title and abstract analysis; six were excluded after the full-text study. Additional eight publications were excluded for other reasons or if they did not apply to the study criteria. Out of the remaining 23 articles, we conducted a quality assessment on five case reports, 11 observational studies out of which three are cross-sectional studies, two are case-control studies, four are prospective cohort studies, and two are retrospective cohort studies.

**Figure 1:**
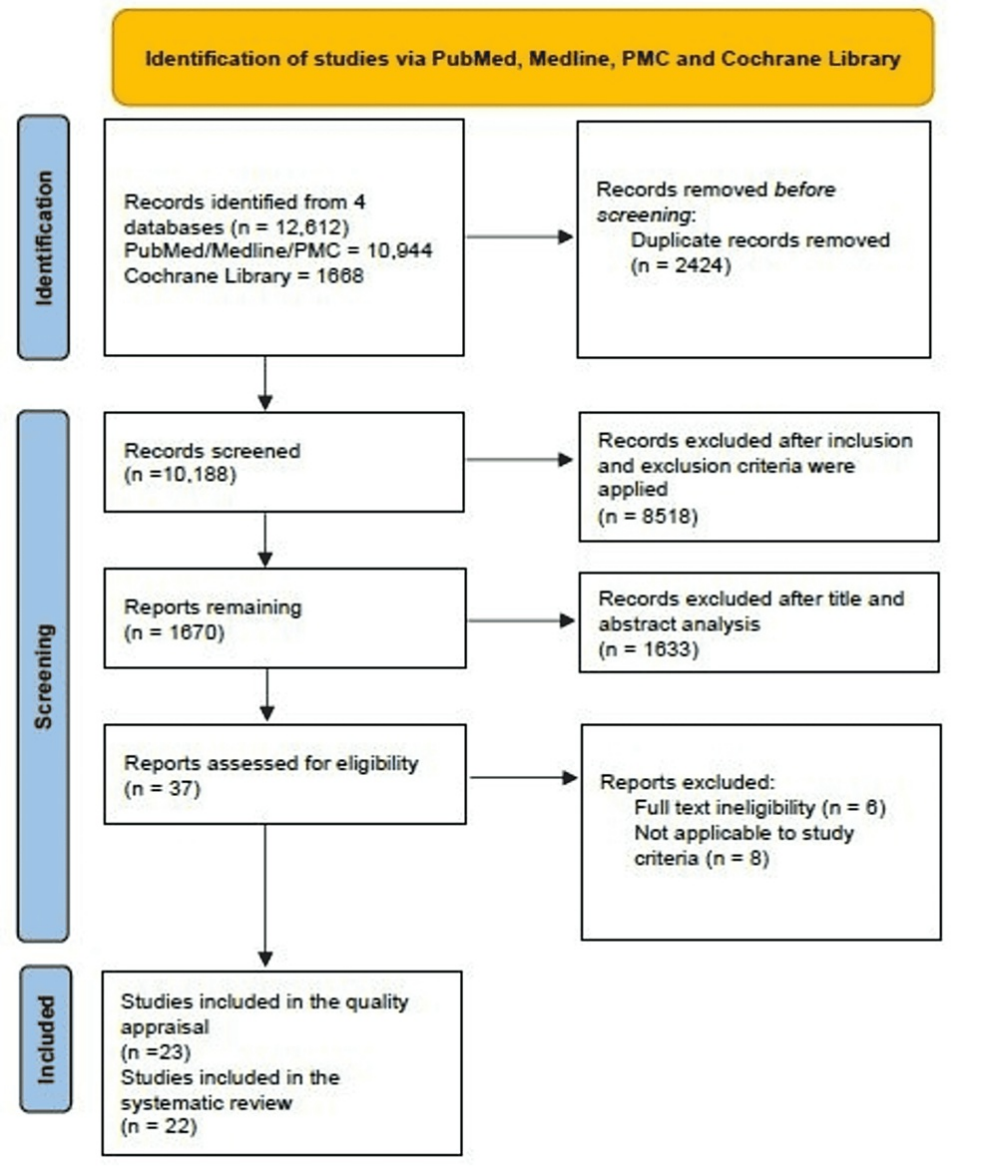
PRISMA flow diagram depicting the article selection process. PRISMA, preferred reporting items for systematic review and meta-analysis

We also reviewed three systematic reviews, three randomized control trials (RCTs), and one narrative review. One RCT was eliminated after quality appraisal due to a low score; 22 publications were included in the study. A complete Preferred Reporting Items for Systematic Reviews and Meta-Analyses (PRISMA) flow diagram is shown below in Figure 1 [12]. Table 3 includes the list of all the publications included in this systematic review. Tables 4-8 below show quality assessments of all the review articles mentioned in this systematic review.

**Table 3:** List of publications included in this systematic review. RCT, randomized control trial

Author	Type of study	Number of participants	Purpose of the study	Results/Conclusion
Cunniffe et al. (2019) [6]	Case report	1	Accidental aspiration of dentures after general anesthesia	Dislodged denture during anesthesia resulting in odynophagia, dysphagia, and recurrent episodes of hemoptysis
Axe et al. (2016) [8]	Observational study (retrospective cohort study)	613	Current dental health professional recommendations and consumer habits in a denture cleaning	The study failed to provide any clear consensus on dental cleansing recommendations among dental healthcare providers
Shedlin et al. (2018) [10]	Observational study (prospective cohort study)	194	Analyze the knowledge and oral health care behaviors among underserved older adults	Underserved older individuals from various cultural backgrounds understand the importance of oral care; however, healthcare disparities might exist among them
Fernandes et al. (2020) [11]	Systematic review		Relationship with oral candidiasis and denture stomatitis in diabetic patients	The rate of oral candidiasis is similar in diabetic and non-diabetics, however, there is a higher rate of denture stomatitis in a diabetic patient compared to non-diabetics
Nakamura et al. (2021) [18]	Case report	1	Unhygienic dentures causing life-threatening infections	Poor denture care in combination with gingival bleeding results in *S.oralis* meningitis.
Derafshi et al. (2017) [19]	Observational study (cross-sectional)	100	Identification of nonoral pathogenic bacteria in the oral cavity of patients with removable dentures	Participants with removable dentures revealed *E. coli*, *K. pneumoniae*, *Enterobacter aerogenes*, *E. cloacae,* and Gram-negative bacilli were found significantly more in the case group than in the control group
Leoney et al. (2020) [20]	Observational study (case-control study)	30	Detection of biofilm *Staphylococcus aureus*, *Viridans streptococcus*, *K. pneumoniae*, and *Escherichia coli*	These biofilms can cause systemic infections that are highly resistant to conventional treatment.
Silva et al. (2016) [21]	Observational study (prospective cohort study)	52	Determine the occurrent and antimicrobial susceptibility of enteric rids and pseudomonas from denture biofilms.	K.pneumoniae, E.coli and E.aerogenes were found on denture biofim. Most enteric bacilli and Pseudomonas species were resistant to amoxicillin and amoxicillin clavulanate. All organisms were susceptible to ciprofloxacin.
Gacon et al. (2020) [22]	Observational study (retrospective cohort study)	279	Coexistence of systemic diseases and inflammation in the oral cavity in edentulous patients.	Systemic diseases in edentulous people using removable devices, and the use of medications for these diseases may actually result in lack of clinical symptoms of oral mycosis.
Kusama et al. (2019) [23]	Observational study (cross-sectional)	71,227	Infrequent denture hygiene and its increased risk of pneumonia.	Infrequent denture cleaning and wearing dentures during sleep was associated with higher incidence of pneumonia.
Linuma et al. (2015) [24]	Observational study (prospective cohort study)	524	Dentures wearing during sleep increases the risk of pneumonia	Overnight denture wearing was independently associated with approx. 2-3-fold higher risk of incidence of pneumonia. Those who wore dentures overnight were more likely to have tongue and denture plaque, gum inflammation, higher levels of circulating interleukins, and positive cultures for *Candida albicans* compared to their counterparts.
Liu et al. (2018) [25]	Systematic review		Incidence of pneumonia among elderly people residing in nursing homes.	It is highly suggested professional care could reduce mortality due to pneumonia in nursing home residents however no high-quality evidence to determine which oral care measures are most effective.
Ribeiro et al. (2019) [26]	RCT	100	Does the implementation of hygiene practices aid in the treatment of denture-related stomatitis?	Initiated oral hygiene practices for oral prosthesis can achieve decreased incidence of denture stomatitis and may also decrease the risk of cardiac diseases
Maciag et al. (2014) [27]	Observational study (case-control study)	44	Denture-related stomatitis and its associated with endothelial dysfunction	Systemic inflammation may affect vascular dysfunction. Increased cardiovascular risk has been shown to increase caries as well as endodontic infection and increasing denture-related stomatitis.
Kamboj et al. (2021) [28]	Case report	1	Aspiration of dentures after an ischemic stroke	Inaccurate clinical evaluation and premature advancement of diet led to aspiration of the denture and its resulting aspiration pneumonia and death.
Murray et al. (2014) [29]	Case report	1	Aspiration and obstruction of dentures in the bronchus	Endobronchial actinomycosis is found in association with foreign body aspiration or necrotizing pneumonias.
Slade et al. (2015) [30]	Case report	1	Aspiration of dentures following cerebral infarction	Dysphagia, dysphonia, and sore throat can be due to accidental swallowing of dentures
Yen et al. (2015) [31]	Observational study (cross-sectional)	277	Impact of removable dentures on oral health-related quality of life	Elderly patients using removable dentures suggest high satisfaction in patient oral health-related quality of life
Wong et al. (2019) [32]	Systematic review		To review oral health levels, oral health-related quality of life among older institutional residents	Review of evidence-based knowledge on oral health and its associated factors among the elderly. Discussion of modifiable factors such as social inequality, oral healthcare accessibility, and providing quality nursing services.
Cankaya et al. (2020) [33]	An observational study (prospective cohort study)	553	Association between denture care, oral hygiene, and periodontal status of elderly patients using removal dentures	Some 51.1% of patients with poor denture cleaning report that they have not been provided information about denture care. The success of denture maintenance might depend on elderly patients' knowledge of denture care, hygiene habits, and ultimately their motivation.
Northridge et al. (2020) [34]	Narrative review		Disparities in oral healthcare	Individuals with low income, uninsured, members of racial/ethnic minorities, immigrants, and rural residents are more likely to have poor oral health than those with better access to quality oral healthcare.
Weintraub et al. (2018) [35]	RCT	219	Improving oral hygiene among nursing home residents	The oral care intervention implemented among nursing home residents improved gingival inflammation, reducing dental plaque and overall denture cleanliness.

**Table 4:** Cochrane assessment tool for RCTs. RCTs, randomized control trials

Cochrane assessment tool for RCTs (low risk/high risk/uncertain)	Ribeiro et al. (2019) [26]	Weintraub (2018) [35]
Random adequate sequence generation	Low risk	Low risk
Allocation concealment used	Low risk	Low risk
Blinding	Low risk	Unclear
Concurrent therapies similar	Unclear	Low risk
incomplete outcome data addressed	Low risk	Unclear
Uniform and explicit outcome	Low risk	Unclear
Free of selective outcome	Unclear	Low risk
Free of other bias	Low risk	Low risk
Our evaluation (out of 8)	6/8 = 75%	5/8 = 62.5%

**Table 5:** JBI checklist for case reports. JBI, Joanna Briggs Institute

JBI checklist for case reports [Yes/No/Not applicable (N/A)]	Cunniffe et al. (2019) [6]	Nakamura et al. (2021) [18]	Kamboj et al. (2021) [28]	Murray et al. (2014) [29]	Salde et al. (2015) [30]
Patient demographics	Yes	Yes	Yes	Yes	Yes
Patient’s history	Yes	Yes	Yes	Yes	Yes
Current clinical condition	Yes	Yes	Yes	Yes	Yes
Diagnostic test or assessment	Yes	Yes	Yes	Yes	Yes
Intervention/treatment procedure	Yes	Yes	Yes	Yes	Yes
Post-intervention condition	Yes	Yes	Yes	Yes	Yes
Adverse events	N/A	Yes	N/A	N/A	N/A
Takeaway lessons	Yes	Yes	Yes	Yes	Yes
Our evaluation (out of 8)	7/8 = 88%	7/8 = 88%	7/8 = 88%	7/8 = 88%	7/8 = 88%

**Table 6:** AMSTAR criteria for systematic reviews. AMSTAR, Assessment of Multiple Systematic Review

AMSTAR criteria for systematic reviews (Yes, NO, Uncertain)	Fernandes et al. (2020) [11]	Liu et al. (2018) [25]	Wong et al. (2019) [32]
A priori design	Yes	Yes	Yes
Duplicate study selection and data extraction	Yes	Yes	Yes
A comprehensive literature search performed	Yes	Yes	Yes
Status of publication (grey literature) used as an inclusion criterion	Yes	Yes	No
List of studies	Yes	Yes	Yes
Characteristics of included studies	No	Yes	Yes
Scientific quality	Yes	Yes	Yes
Formulation of conclusion	Yes	Yes	Yes
The method used to combine findings	Yes	Yes	Yes
Likelihood of publication bias	No	No	No
Conflict of interest	No	No	Yes
Our evaluation (out of 11)	Score of 8/11	Score of 9/11	Score of 9/11

**Table 7:** SANRA checklist for narrative review articles. SANRA, Scale for the Assessment of Narrative Review Articles

SANRA (each out of 0,1, or 2)	Northridge et al. (2020) [34]
Justification of the article's importance for the readership	2
Statement of concrete aims or formulation of questions	2
Description of the literature search	0
Referencing	2
Scientific reasoning	2
Appropriate presentation of data	1
Total score	9/12 = 75%

 

**Table 8:** Newcastle-Ottawa quality assessment for observational studies.

Newcastle-Ottawa quality assessment for observational studies	Representative of the exposed cohort	Selection of the non-exposed cohort	Ascertainment of exposure	Demonstration that the outcome of interest was not present at the start of the study	Comparability of cohorts based on the design	Assessment of outcome	Was followed up long enough for outcomes to occur	Adequacy of follow-up cohorts	Our evaluation
Axe et al. (2016) [8]	*	Nil	*	*	*	*	Nil	Nil	Score 5/8
Shedlin et al. (2018) [10]	*	Nil	*	Nil	*	*	*	Nil	Score 5/8
Derafshi et al. (2017) [19]	*	Nil	*	*	*	*	Nil	Nil	Score 5/8
Leoney et al. (2020) [20]	*	Nil	*	Nil	*	*	*	*	Score 6/8
Silva et al. (2016) [21]	*	Nil	*	*	*	*	*	*	Score 7/8
Gacon et al. (2020) [22]	*	Nil	*	*	*	*	Nil	Nil	Score 5/8
Kusama et al. (2019) [23]	*	Nil	*	Nil	*	*	*	*	Score 6/8
Linuma et al. (2015) [24]	*	Nil	*	*	*	*	*	*	Score 7/8
Maciag et al. (2014) [27]	*	*	*	Nil	*	*	Nil	Nil	Score 5/8
Yen et al. (2015) [31]	*	Nil	*	Nil	**	*	*	*	Score 7/8
Cankaya et al. (2020) [33]	*	Nil	*	*	*	*	Nil	*	Score 6/8

Discussion

Effects of Biofilm Formation on Removable Dentures 

Oral health is crucial for various aspects of life, including eating, talking, self-esteem, social interaction, and freedom from pain. Poor oral care can result in plaque formation, gingival irritation, halitosis, dental caries, periodontal diseases, tooth loss, and systemic illnesses such as pneumonia, complications from diabetes, and even death.

A case report by Nakamura et al. discussed an 81-year-old male who presented with symptoms of meningitis, including fever, headaches, and neck stiffness. Before this, he had transient gingival bleeding 10 days before admission. The patient had full dentures with no dental care for over 10 years and showed +marked plaque adhesions. The patient’s blood culture revealed *Streptococcus oralis* indicating a possible path of infection that could lead to odontogenic bacteremia. Poor oral hygiene resulted in gingival bleeding, leading to *S. oralis* meningitis [18].

A cross-sectional study by Derafshi et al. with 100 participants discussed the identification of nonoral pathogenic bacteria in the oral cavity of patients with removable dentures. Many pathogens were identified in removal dentures, such as *Escherichia coli*, *Klebsiella pneumoniae*, *Enterobacter cloacae*, and Gram-negative bacilli (Enterobacteriaceae and nonfermenting) were significantly more in denture wearers compared to the control group without denture usage. These pathogenic species may cause infections that can disseminate to other body parts. Oral health measures should be implemented to reduce the risk of cross-infection in individuals with removable dentures [19].

An observational study by Leoney et al. with 30 denture users was selected. Biofilm-forming *Staphylococcus aureus*, *Viridans streptococcus*, *Klebsiella pneumoniae*, and *Escherichia coli* were isolated from their dentures. These biofilms cause planktonic bacteria to propagate throughout the body, potentially leading to systemic disorders that are resistant to traditional therapy. This could be owing to the biofilm's natural ability to provide medication resistance to currently available antibacterial treatments [20]. 

An observational study by Silva et al. with 52 subjects studied the manifestation and the in vitro antimicrobial susceptibility of enteric rods and pseudomonas on biofilm formed on denture users. All organisms were susceptible to ciprofloxacin, and most species were resistant to amoxicillin and amoxicillin-clavulanate. Therefore, preventive programs for biofilm control are essential in avoiding colonization of dental prostheses and its resulting multidrug-resistant bacterial infections [21].

Another observational study by Gacon and Wieczorek strongly suggested that oral infections can increase the risk of systemic illnesses such as atherosclerosis, coronary heart diseases, chronic obstructive pulmonary disease, stroke, diabetes, rheumatoid arthritis, and many more. The study was conducted with 279 patients; 58% of patients with hypertension revealed no signs of inflammation. The onset of systemic diseases in edentulous patients wearing prosthetic devices and the subsequent use of medications to treat these diseases may lack clinical symptoms of simultaneous oral mucosal fungal infection [22].

Use of Dentures and Its Related Increased Risk of Pneumonia

The relationship between the aspiration of oral bacteria and pneumonia is very heavily supported; the increased incidence of stroke, cognitive decline, and dysphagia further increase the risk of aspiration pneumonia among the aging population. Three studies in our systemic review discussed the use of dentures and their subsequent increase in pneumonia risk. 

A cross-sectional study by Kusama et al. with 71,227 participants studied the relationship between frequency of denture cleaning, literacy rate, smoking status, dementia, stroke, and their association with pneumonia. Pneumonia was more prevalent among the participants who did not clean their dentures daily, especially those older than 75. Among these participants over the age of 75, 2.5% and 4.3% of those who did and did not clean their dentures daily, respectively, experienced pneumonia. Infrequent denture cleaning was significantly associated with the occurrence of pneumonia among those aged older than 75 years (odds ratio, OR = 1.58, 95% confidence interval, CI = 1.15-2.17). The same was not observed in participants less than 75 years of age. 

In addition, participants with less than nine years of education had about 42.4% experienced pneumonia. A higher proportion of reported cases were also seen among participants with low socioeconomic status. This study concludes that periodic denture cleaning was associated with an increased incidence of pneumonia in older individuals; daily cleaning and proper instructions from dental professionals are further necessary for pneumonia prevention [23].

An observational study by Linuma et al. with 524 subjects was evaluated to determine if those who wore their dentures during sleep increased the risk of pneumonia in elderly patients. Forty-eight events were associated with pneumonia (20 deaths and 28 acute hospitalizations). Denture use during sleep was associated with an approximately 2.3-fold higher risk of pneumonia (hazard ratio 2.45;95% CI, 1.23-4.51). This study concluded that those who wore dentures during sleep were at higher risk of tongue and denture plaque, gum inflammation, positive culture for Candida albicans, and higher circulating interleukin-6 (Il-6). In addition, those who developed pneumonia were more likely to have swallowing difficulties, a habit of sleeping with dentures, disability involving activities of daily living (ADL), cognitive impairment, a lower body mass index (BMI), a history of respiratory disease and stroke, or low albumin level [24]. 

A systematic review by Liu et al. consisting of 3905 participants was conducted to assess the effects of oral hygiene measures in preventing nursing home-acquired pneumonia in residents of long-term care facilities. The studies compared patients who received professional oral care and usual oral care. The study commented that professional oral care might reduce pneumonia-associated death by 60% more than usual oral care at a 24-month follow-up. Another study in the review showed that fewer participants with pneumonia in the professional care group (21 of 184) than in the usual oral care group (34 of 182) (risk ratio, RR 0.61, 95% CI 0.37-1.01) [25].

Denture-Related Stomatitis and Its Effects

A systematic review by Martorano-Fernandes et al. showed diabetic patients had a similar incidence of developing oral candidiasis to non-diabetic patients [OR 1.40 (0.96;2.04), p = 0.008, I2 = 94%]. Higher chance of denture stomatitis is present in diabetic patients in comparison to non-diabetic patients [OR 1.92 (1.42;2.59) p < 0.0001, I2 = 0%] [11].

Another randomized controlled trial by Ribeiro et al. with 100 patients divided into four groups of 25 patients, each with different oral hygiene protocols, was conducted to determine. Group-1 - brushing the palate with a soft brush and prosthesis immersion in 0.25% sodium hypochlorite solution. Group-2 - brushing the palate with a soft brush and prosthesis immersion in 0.15% triclosan solution. Group-3 - brushing the palate with a soft brush and prosthesis immersion in citric acid. Group-4 - prosthesis immersion and brushing the palate with citric acid and a soft brush. Cardiac variability and blood pressure were recorded in the control period and after the treatment of denture stomatitis through the oral hygiene protocols. Denture stomatitis has been associated with variation in blood pressure and endothelial dysfunction that may lead to the development of cardiovascular diseases [26].

There is also an observational study by Maciąg et al. with 44 patients evaluated if denture-related stomatitis is associated with endothelial dysfunction in elderly patients with dentures. Diabetes and smoking increase the risk of both denture-related stomatitis and cardiovascular disease. In the study, 60% of patients wearing dentures are associated with a reduction of endothelial dysfunction function, which correlates with the development of coronary artery diseases and may even predict future cardiovascular events [27].

Complications of Accidental Aspiration or Ingestion of Removal Dentures 

Five case reports discuss the complications of ingestion of dentures. A case report by Kamboj et al. revealed a metallic foreign body in the pharynx at the level of the epiglottis after the patient in the report suffered an acute ischemic event. This particular patient’s hospital course was complicated by septic shock due to aspiration pneumonia, requiring the need for broad-spectrum antibiotics and mechanical ventilation. Unfortunately, due to the complications, the patient passed away [28].

Another case report by Murray et al. discussed a 65-year-old man with a 12-month history of recurrent respiratory infections. Imaging studies showed obstruction of the left main bronchus with prominent granulation tissue. Endobronchial sampling discovered actinomycosis, which is rare and uncommon but can be seen with foreign body aspiration or necrotizing pneumonia. Actinomycosis may falsely present as bronchogenic malignancy and tuberculosis. The presence of this organism in the airway, according to the report, should prompt a comprehensive examination of a foreign body [29].

A case report by Slade and Larsen about a 75-year-old female presented with symptoms of dysphagia, dysphonia, and sore throat following a cerebrovascular accident. The patient failed bedside swallow evaluation, and a nasogastric tube was inserted for medications and early nutrition. After multiple days of hospitalization with no improvement in her symptoms of dysphagia and dysphonia, the patient expectorated a plastic item which happens to be her denture and prosthetic tooth. Imaging studies failed to show this foreign object. This study concluded that the oral cavity should be thoroughly examined for missing teeth or dentures when patients present with dysphagia or pain in their throat [30].

Yet another case report by Cunniffe is about a 72-year-old man who presented with odynophagia, dysphagia, and hemoptysis six days after a minor surgery under anesthesia. Imaging studies and flexible nasendoscopy revealed a metallic semicircular object. After discovering and removing the dentures, he developed aspiration pneumonia and hemoptysis due to granulation tissue formation. This study concluded that any dental prosthesis should be documented before and after any procedure, and the perioperative strategy should be communicated to all members of the operating room [6].

Quality of Life and Role of Oral Hygiene With the Use of Removable Dentures Among Elderly

A cross-sectional study was conducted by Yen et al. in Taiwan with 277 elderly patients. The impact of removable dentures on oral health-related quality of life was studied. Factors related to denture use and oral health-related quality of life -- physical function, psychosocial function, pain, and discomfort were studied. Some 18.4% of patients suffered loose dentures, and 10.4% noted the presence of an oral ulcer. Subjects with oral ulcers and those who perceived loose dentures were more likely to state a poorer quality of life. Results also showed the larger number of remaining natural teeth positively affected the quality of life. The study concludes that denture satisfaction was a strong predictor determining the quality of life in the elderly wearing removable dentures [31].

A systematic review by Wong et al. identified factors affecting the oral quality of life among elderly institutionalized patients. The study showed that residents with dentures often were found to have difficulty cleaning their dentures, relaxing, or sleeping. They also reported discomfort eating in front of others and limitations in the variety of foods they can consume, affecting their quality of life. Dental health has an impact on an individual’s physical and emotional well-being. Malnutrition is common in individuals with poor oral health, affecting their quality of life. Residents with a lower educational level had a reduced quality of life. Females, socially isolated individuals, and those with cognitive disabilities may all have a lower quality of life [32].

An observational study by Shedlin et al. with 194 discussed how underserved elderly patients from different cultural backgrounds understand their oral hygiene and its effects on their social lives. Patients report that dentures were painful, preventing them from using them; some report that they assumed they are not required to seek dental care anymore as they have dentures. The study commented that participants were experiencing problems affording copayment, and several co-morbidities preventing multiple visits involved with treatment and restoration. In addition, there was also a lack of affordable dental facilities and confusion about Medicaid coverage [10]. 

Another observational study by Cankaya et al. with 533 participants studied the association between denture care and oral hygiene habits in patients with removable dentures. Some 54.1% of the participants reported not receiving any information about denture care. Some 41.25% of patients with denture stomatitis had poor denture hygiene habits. Some 50% of patients with poor denture hygiene have a smoking history. The study reported a strong relationship between age of dentures, overnight denture usage, frequency of smoking, denture stomatitis, and frequent denture cleaning (p < 0.05). It concluded that the success of denture maintenance might depend on elderly patients' knowledge of denture care, hygiene habits, and, ultimately, their motivation [33].

Disparities in Oral Healthcare and Its Limitations 

A narrative review by Northridge et al. stated that severe states have reduced or even discontinued Medicaid dental coverage. Medicaid enrollees often have difficulty finding dental providers accepting Medicaid, as only 20% of dentists nationwide accept Medicaid. Barriers to participation in Medicaid programs by dental facilities are primarily due to lengthy payment wait times, low reimbursement rates, several missed appointments, and cumbersome administrative requirements. Individuals with low income, uninsured, members of racial/ethnic minorities, immigrants, and rural residents are more likely to have poor oral health than those with better access to quality oral health care [34]. 

A previously mentioned observational study by Shedlin et al. discussed that the Medicare program does not cover basic dental care for individuals over 65. They are frequently unable to afford the essential preventive and rehabilitative therapies. Similarly, African American and Hispanic older individuals are more likely than white older adults to have poor self-rated oral health and untreated dental disease. Over one-third (34%) of those living below the federal poverty level are edentulous, compared to only one-eighth (13%) of those living above the federal poverty line among US adults aged 65-74 years. Improving patients' oral health literacy at the individual level and strengthening the cultural competency of oral health practitioners at the interpersonal level may be effective in advancing oral health equity [10].

Recommended Evidence-Based Guidelines on Denture Hygiene Practices

A previously discussed observational study by Axe et al. mentions recommendations made by dental professionals for individuals using dentures. Some four products of dental routines were studied (toothpaste, denture cleanser tablets, mouthwash, and soap and water). More than 10% of dental professionals did not provide primary cleaning recommendations. According to the study, dentures should be cleaned every day, ideally overnight, with a nonabrasive denture solvent. After soaking and brushing with a denture cleanser, they should always be properly rinsed before inserting into the mouth. Denture tablets were more generally advised in developed countries, whereas toothpaste was recommended in developing countries. Denture tablets were used less frequently than toothpaste, water, and mouthwash due to added cost and inaccessibility. More than 75% of denture wearers said they used denture cleansing tablets for more than five minutes, compared to fewer than two minutes for soap and toothpaste. The findings show a difference between dental professionals’ recommendations and denture users’ oral care habits, and there is no consensus on the best denture cleaning procedures [8].

A randomized control trial by Weintraub et al. was a study conducted at nursing home facilities to determine if oral care practices by nursing staff could potentially improve residents’ oral hygiene and denture outcomes. “Mouth Care Without a Battle” is a collection of techniques and products to clean and protect the teeth, tongue, gums, and dentures. Plaque index (range 0-3), gingival index (0-4), and denture plaque index (0-4) were studied among the interventional group and control group after a 24-month follow-up duration with a lower score indicating better oral condition. After the study period, the intervention group had a significant improvement in oral and denture hygiene compared to the control group (p < 0.05), with mean changes in indices of plaque index (0.44), gingival index (0.55), and denture plaque index (0.67) points lower in the intervention group than the control group [35].

Limitations

The limitation of this study is that most of the articles reviewed were either observational or case reports. Analysis of English-only articles might have limited numerous other studies conducted in other languages regarding proper denture care and its complications among users. We have also limited our selection of publications to only cases and studies with patients over 65; this could have limited our analysis further. 

## Conclusions

This systematic review analyzed inappropriate denture use and poor oral hygiene in the elderly population using removable dentures. Our study highlighted the complications associated with improper use of dentures. Through our review, we have seen pathological biofilm formation on dentures, life-threatening cases of pneumonia, denture-related stomatitis, and accidental ingestion/aspiration of dentures amongst elderly populations. Most of these complications can be easily prevented with proper guidance and education. However, it is unfortunate that the majority of the elderly population and their caregivers are not adequately educated about proper cleaning techniques by their dental providers. In addition, there is also a wide inequality of access to dental care by patients on Medicaid or individuals of minority groups, complicating care even further. Oral hygiene and well-being significantly impact an individual’s social, physical, and psychological status. Therefore, addressing these issues is important to society more than ever before. In the future, a further detailed study should be conducted as no clear consensus exists in terms of the best practice of denture cleaning methods. In addition, measures should be initiated to encourage regular dentist appointments and increase accessibility among members of low socioeconomic status, low literacy rate, and minorities.
